# Effect of Freezing for up to 120 Days on the Physicochemical Characteristics of Hamburgers Made from Botucatu Rabbit Does Slaughtered at Different Ages

**DOI:** 10.3390/ani15121805

**Published:** 2025-06-19

**Authors:** Erick Alonso Villegas-Cayllahua, Daniel Rodrigues Dutra, Ana Veronica Lino Dias, Thamiris Daiane Domenici, Leandro Dalcin Castilha, Hirasilva Borba

**Affiliations:** 1 Department of Agricultural and Environmental Biotechnology, Faculty of Agriculture and Veterinary Sciences, São Paulo State University, Jaboticabal 14884-900, Brazil; danielrdutra@hotmail.com (D.R.D.); ana.lino@unesp.br (A.V.L.D.); thamiris.domenici@unesp.br (T.D.D.); hirasilva.borba@unesp.br (H.B.); 2 Department of Animal Science, State University of Maringá, Maringá 87020-900, Brazil; ldcastilha@uem.br

**Keywords:** age, carcass, cooking loss, lipid oxidation, moisture, *Oryctolagus cuniculus*, tenderness, storage

## Abstract

This study evaluated the effect of freezing for up to 120 days on the physicochemical and technological properties of hamburgers made from Botucatu rabbit does slaughtered at 3, 12, and 24 months of age. The parameters were evaluated as follows: surface color (L*, a*, b*), pH using an insertion pH meter, cooking loss using a grill, storage loss based on weight differences, shear force in cooked samples using a texture analyzer, shrinkage percentage, chemical composition (moisture, protein, lipids, and ash), and lipid oxidation, determined by measuring the concentration of malondialdehyde in the burgers at different storage intervals (0, 60, and 120 days) under freezing conditions (−18 °C). The results indicated that increased storage time and animal age reduced tenderness and increased lipid content (*p* < 0.05). Burgers made from younger does showed higher levels of lipid oxidation. Age also influenced color (greater redness and lower lightness in older animals) and chemical composition, with older does producing burgers with higher protein and lower moisture and mineral content. However, all samples remained within the limits established by Brazilian legislation. This study recommends using meat from does of different ages for hamburger production, as all variations met the required legal standards.

## Simple Summary

Breeding rabbits, especially females, are intended for human consumption after their production cycle. However, since the quality of the meat from these older animals is different compared to that from younger rabbits, there are ways to add value, such as the preparation of processed products, such as hamburgers. In this scenario, the objective of this study was to evaluate the effect of freezing for up to 120 days on the physical, chemical, and technological characteristics of hamburgers made with meat from Botucatu rabbits, from animals raised at different ages. The quality of rabbit hamburgers produced at different ages does not change during four months of frozen storage. Based on the results, it is recommended to use meat from rabbits of different ages to prepare hamburgers.

## 1. Introduction

Rabbit meat is considered of high quality due to its characteristics that align with current consumer preferences [1], such as low concentrations of lipids, saturated fatty acids, and cholesterol [1,2], while offering high levels of protein [3,4]. This could be a good source of animal protein for athletes, such as bodybuilders, who require lean, high-protein meat for muscle development [3].It has also been recorded that rabbit meat has low levels of sodium, which is closely related to cardiovascular problems and hypertension [5]. This makes it a recommended choice for patients with cardiovascular issues and hypertension [6].

These characteristics have encouraged researchers and producers to develop strains suited for high-quality meat production. One example is the Botucatu strain, which exhibits excellent reproductive traits (e.g., larger litter size) and productive performance (e.g., achieving 5 kg of live weight). It remains the only commercial strain currently produced in Brazil [7].

However, one of the main barriers to its consumption is the limited variety of products available in the market, with the primary method of selling being the whole carcass or cuts [1]. Processing rabbit meat (e.g., aging, salting, and producing processed products) is one of the key strategies in the industry to make it more accessible to consumers [8,9].

The production of processed products is also a way to utilize meats that typically show darker color and coarser texture, such as those from older animals [10,11]. This is a good alternative in production systems with high replacement rates, due to the slaughter of rabbits based on age, as observed in rabbit farming systems with replacement rates of up to 120% per year for females, which represent a good part of the breeding stock on farms. Therefore, the fate of these females after the completion of their reproductive cycle is very important, due to the maintenance costs of these females for rabbit farmers [12], in addition to providing a certain economic return after the animal is discarded from the farm.

Furthermore, the processed food industry recommends the maximum storage time for each product, depending on its nature and conservation method, aiming to maintain stable physical, chemical, and sensory characteristics for as long as possible. In the case of hamburgers, there are some recommendations for preserving hamburgers, such as in the United States, where it has been recorded that the United States Department of Agriculture (USDA) recommends a maximum freezing time (−18 °C) of 120 days, in order to maintain product quality [13].

The reason for this recommended time is that, although freezing is an efficient way to preserve the intrinsic characteristics of the product for longer, as storage time increases, oxidation processes occur that can jeopardize its quality, especially in meats that present high concentrations of PUFAs, such as rabbit meat [14].

Although there are some studies evaluating the effect of the age of the rabbit on the quality of the meat, there are no studies evaluating the interaction of the effect of the age of the female rabbit and the storage time on the quality of derived products, such as the case of hamburgers, which are one of the most consumed derived products today due to their easy preparation and low purchase price [15].

Therefore, the aim of this study was to evaluate the effect of freezing for up to 120 days on the physicochemical and technological characteristics of burgers made from Botucatu breed rabbit meat, sourced from animals culled at different ages.

## 2. Materials and Methods

This work was conducted at the Rabbit Farming Section and the Laboratory of Animal Origin Food Analysis Laboratory (LaOra) at the Faculty of Agrarian and Veterinary Sciences of the São Paulo State University (UNESP), Jaboticabal Campus, São Paulo, Brazil (21°08′ S, 48°11′ W, 583 m altitude), with approval from the Animal Ethics Committee (CEUA) of this institution (protocol no. 5431/20). The slaughter procedure was carried out in compliance with current regulations related to the industrial and health inspection of products of animal origin [16], as well as the provisions established by European Regulation (EC) No. 1099/2009 [17].

### 2.1. Sample Collection and Experimental Procedure

Thirty rabbits from the Botucatu lineage, of the same farm and having undergone the same management conditions, were used in the experiment, distributed into 3 experimental groups (T1: young does, 3 months old; T2: does, 12 months old; T3: does, 24 months old), with 15 does per group. They were slaughtered at a commercial rabbit slaughterhouse inspected by the Federal Inspection Service (SIF).

After slaughter, the carcasses were kept in a cold chamber (4 °C) for 24 h and then placed in a freezing tunnel (−18 °C). They were then transported by truck with temperature control at −18 °C to the Laboratory of Animal Origin Food Analysis (LaOra), at the Department of Agricultural and Environmental Biotechnology, FCAV/UNESP, Jaboticabal Campus, for deboning the carcasses.

After deboning, the raw meat and other ingredients were weighed separately on an analytical digital scale (Table 1). The rabbit meat and chicken skin were processed through a meat grinder with a 5 cm disc.

The hamburger mixtures underwent a homogenization process, were mixed in a rotating bowl for 10 min, and were removed from this equipment at temperatures lower than 14 °C.

The mixture resulting from the previous step was divided into portions of 100 g and then shaped into molds of 11.2 cm. The product was frozen at −20 °C. Ten repetitions per treatment were made, with each repetition consisting of a plastic package containing 3 hamburgers, being considered as an experimental unit. The first burger will be used to evaluate the color and pH, the second burger will be used to evaluate the cooking loss (CL), storage weight loss (SWL), and shear force (SF), and the third burger will be used for the chemical composition.

Part of the samples (n = 10 repetitions of 3-month-old Botucatu does; n = 10 repetitions of 12-month-old Botucatu does; n = 10 repetitions of 24-month-old Botucatu does) were evaluated on day 0 (beginning) and subsequently at the end of each proposed freezing period (60 and 120 days). The physicochemical analyses were conducted on the hamburgers made from the meat of Botucatu does of different ages (n = 10) after thawing in refrigeration (4 ± 2 °C) in an incubator (Eletrolab EL101/3 116 250W, Eletrolab, São Paulo, SP, Brazil), with 120 days being the maximum recommended time for using hamburgers stored under freezing conditions [13].

### 2.2. Physicochemical Analysis

#### 2.2.1. Meat Color

A Minolta Chrome Meter model CR-400 was used, which employs the CIELAB system [18], with the L*, a*, b* scale of the CIELab system, using illuminant D65, an observation angle of 10°, and a 30 mm cell aperture. Color measurements were performed in triplicate on the surface of the thawed burger (4 °C).

#### 2.2.2. pH, Cooking Loss (CL), Storage Weight Loss (SWL), and Shear Force (SF)

The pH was determined in triplicate using a digital pH meter (Testo model 205) by inserting the electrode into the hamburger. Calibration was carried out according to the manufacturer’s recommendations, with the pH meter calibrated using two buffer solutions, pH 4.00 ± 0.2 and pH 7.00 ± 0.2, both from the same company (Dinâmica Química Contemporânea Ltda, Indaiatuba, SP, Brazil). The pH meter automatically compensated for temperature.

The cooking loss of the hamburger (CL) was determined using the methodology described by Mello et al [19]. The frozen hamburgers were weighed on an analytical scale. Then, all the products were grilled together for 12 min on an electric grill (George Foreman GBZ80, Lake City, FL, USA) while still frozen, for 6 min per surface. After cooling to room temperature, they were weighed again to determine the CL and expressed as a percentage, using the following formula:

*CP* (%) = (*Initial weight* − *Final weight*) × 100/*Initial weight*

The shrinkage percentage (SP) of the hamburger was evaluated by measuring the diameter of the still-frozen hamburger’s cross-section in three distinct regions using a digital caliper, in triplicate. After cooking, the diameter was measured again and expressed as a percentage (%).

*SP* = (*Initial area* − *Final area*) × 100/*Initial area*

The weight loss during storage (SWL) was assessed by the difference between the initial weight (g) of the hamburger at time 0 (beginning) and the weight of the defrosted hamburger (g), expressed as a percentage (%).

*SWL* (%) = (*Initial weight* − *Final weight*) × 100/*Initial weight*

Tenderness was assessed using a texture analyzer (TAXT2i, Stable Micro Systems) with a Warner–Bratzler device in triplicate, and the values were expressed in Newtons (N).

#### 2.2.3. Chemical Composition and Lipid Oxidation

The chemical composition was determined through the analysis of the moisture percentage (method 950.46), protein (method 977.14), and mineral matter (method 920.153), according to procedures outlined by the Association of Official Analytical Chemists [20]. The concentration of total lipids was measured using the methodology proposed by Bligh and Dyer [21]. The analysis was performed in duplicate.

Lipid oxidation was determined in duplicate, using the thiobarbituric acid reactive substances (TBARS) test, with the result expressed in mg of malondialdehyde/kg of the sample; following the methodology described by Vyncke et al. [22], 5 g of ground samples were used for extraction using trichloroacetic acid, and then 5 mL of thiobarbituric acid was added and placed in a water bath (100 °C) for 45 min. After the coloring reaction, the reading was performed at a wavelength of 532 nm.

### 2.3. Statistical Analysis

An entirely randomized design was used in a 3 × 3 factorial scheme (age × storage time), consisting of Botucatu does of three different ages (3, 12, and 24 months) vs. three storage times (beginning, 60, and 120 days), with 10 repetitions for each treatment. The experimental unit consisted of a package made up of three hamburgers, each of which was intended for the performance of different physical-chemical analyses.

The results were analyzed using the General Linear Models procedure of the Statistical Analysis System (SAS Institute Inc., Cary, NC, USA). The Shapiro –Wilk test was used to assess the normality of the results. The data were subjected to analysis of variance (ANOVA) and compared using the Tukey test, with significance set at *p* < 0.05.

## 3. Results and Discussion

There was no interaction (*p* > 0.05) between the age and the storage time on the parameters of lightness (L*), redness intensity (a*), and yellowness intensity (b*) of burgers made with meat from Botucatu does of different ages stored at −18 °C for up to 120 days (Table 2).

The age of the female influenced the color of the burgers, with burgers made from the meat of older does (24 months old) showing lower (*p* < 0.05) lightness (L*) (70.8) compared to those made from younger does (3 months old) (71.7). This difference was likely related to pH values closer to the isoelectric point (5.3), which weakens the bond between protein and water molecules, leading to a decrease (*p* < 0.05) in moisture content in burgers made from older Botucatu does. The amount of water in the burger is a key factor influencing lightness [23], with similar results observed in fresh rabbit meat regarding age [9], as well as in other species such as broilers [24].

The burgers made from the meat of adult does (12 and 24 months old) exhibited higher (*p* = 0.007) values of redness (a*) (5.8 and 5.8, respectively) compared to those made from the meat of young does (3 months old) (5.5). This could be related to the fact that, generally, as the animal’s age increases, there is an increase in myoglobin concentrations in the muscle [25,26].

The storage time led to a decrease (*p* = 0.049) in the redness values (a*), possibly due to the increase in lipid oxidation in the burgers, which raises the concentrations of free radicals. This type of oxidation is related to the oxidation of product pigments, such as myoglobin, forming metmyoglobin [27,28,29].

The storage time significantly increased the b* values (*p* = 0.001), with samples frozen for 60 and 120 days reaching higher b* values (11.7 and 11.0, respectively) compared to refrigerated burgers at the initial stage (9.98). Other studies have also reported similar results, likely due to increased brown coloration resulting from myoglobin oxidation, which converts myoglobin into metmyoglobin [30].

There was no interaction (*p* > 0.05) between the rabbit age and storage time on cooking loss (CL), shrinkage percentage (SP), and storage weight loss (SWL) in burgers made with meat from Botucatu does of different ages, stored at −18 °C for up to 120 days (Table 3).

There was no effect (*p* > 0.05) of storage on cooking loss, shrinkage percentage of the burger, and weight loss during storage.

Burgers made with meat from older Botucatu does (12 and 24 months of age) showed lower cooking losses (42.02% and 42.05%, respectively) compared to those made from younger does (3 months of age) (43.75%). This could be related to the intrinsic technological characteristics of raw meat, as it has been observed in some species that meat from older animals tends to show lower cooking losses [11].

The shrinkage percentage of the burger was not influenced by the age of the doe (*p* > 0.05), which is interesting from a technological perspective for using meat from breeding animals, as it is an important quality indicator of the burger [31]. Greater protein denaturation would lead to higher water loss during cooking, causing greater mass loss and a reduction in the cooked burger’s diameter [32].

Regarding weight loss during storage, burgers made from 12-month-old does lost less weight (9.34%) (*p* = 0.001) compared to those made from younger (3 months old) and older (24 months old) does, which lost 10.42% and 10.40%, respectively. This finding holds great importance because water loss during freezing can affect other quality attributes such as tenderness, color, and flavor [33].

There was an interaction (*p* < 0.05) between the age of the doe and storage time on the pH and shear force (SF) of burgers made from the meat of Botucatu does of different ages, stored at −18 °C for up to 120 days (Table 4).

The pH values were higher (*p* = 0.008) in burgers made with meat from 3-month-old does compared to meat from older does (12 and 24 months of age). This is likely because meat from older rabbit does typically has lower pH values than meat from younger does [34]. Given that pH is an indicator of shelf life, the values observed in this study did not exceed the maximum limit defined for meat products considered optimal for human consumption (7.40) [35].

The burgers made from the meat of 24-month-old does were less tender (*p* < 0.05) compared to those made with meat from younger does (3 and 12 months), particularly in the samples frozen for 60 and 120 days. This difference could be attributed to the reduction in soluble collagen percentages in meat as the age of the animal increases [26,36].

Additionally, the storage time increased (*p* < 0.05) the hardness of burgers made from the meat of 24-month-old does. This increase is likely due to water loss during storage, as extended freezing causes ice crystals to grow, further damaging the myofibrillar structure, which is lost during thawing [37]. Given that water content significantly influences meat tenderness, as water content promotes lower compressive strength [33], this could explain the observed texture changes.

There was no interaction (*p* > 0.05) between the age and the storage time regarding the protein, mineral matter (MM), and lipid content of burgers made from the meat of Botucatu does of different ages, stored at −18 °C for up to 120 days (Table 5).

According to the Brazilian technical regulations for the identity and quality of hamburgers [38], products must contain a maximum of 25% fat and a minimum of 15% protein to qualify as hamburgers. The data obtained in this experiment, regardless of the doe’s age or storage duration, meet the requirements outlined in Normative Instruction No. 724, issued on 23 December 2022.

Protein concentrations did not vary with storage time (*p* > 0.05); however, the doe’s age significantly influenced protein content. Burgers made from older does (12 and 24 months of age) contained higher protein levels (24.04% and 22.92%, respectively) than those made from younger does (3 months old), which averaged 21.72%. These findings align with the existing literature, which suggests that as animals age, their meat tends to exhibit higher protein content [39]. This characteristic could positively influence consumer acceptance [40].

Mineral matter concentrations also remained unaffected by storage time (*p* > 0.05) but varied with the doe’s age. Burgers prepared from 24-month-old does contained lower mineral matter (2.14%) than those from 3-month-old does (2.21%). Similar trends reported in studies evaluating raw meat composition indicate that the influence of age on chemical composition persists even after processing into hamburgers [10,41].

The doe’s age did not significantly affect lipid concentrations (*p* > 0.05). However, burgers stored for 60 and 120 days showed higher lipid concentrations (11.73% and 10.98%, respectively) than fresh burgers (9.98%). This increase likely results from the loss of hydrophilic compounds during thawing, when water is released as exudate, and from ice crystal formation and growth during storage, which damages the protein structure of the burgers [42]. There was an interaction (*p* < 0.05) between the age and storage time regarding the moisture content and lipid oxidation of burgers made from Botucatu does, stored at −18 °C for up to 120 days (Table 6).

Burgers prepared from the meat of 3-month-old does showed higher moisture percentages than those from 12-month-old does (*p* < 0.001), and this trend continued throughout storage. The lower moisture content in the meat of older rabbits [39] likely explains this difference.

Regarding lipid oxidation, longer storage times consistently increased oxidation levels, regardless of the animal’s age (*p* < 0.001). Oxidative processes occurring during storage likely contributed to this increase. Researchers have reported similar results for frozen burgers [28]. Although freezing efficiently preserves meat products and maintains their intrinsic characteristics with minimal alteration [43], it does not completely prevent oxidative reactions [44].

Burgers prepared from the meat of younger does (3 months old) exhibited higher initial lipid oxidation levels (1.06 mg MDA/kg) compared to those from 12- and 24-month-old does (0.73 and 0.91 mg MDA/kg, respectively). However, lipid oxidation levels measured during storage in this study remained below the maximum recommended limit of 2.00 mg MDA/kg [45].

This finding holds relevance for the industry, as it suggests that the hamburgers—regardless of storage duration or doe age—did not develop rancid characteristics. Typically, when meat products exceed this threshold, they begin to exhibit detectable rancid flavors and odors, which may lead consumers to reject the product [27]. Furthermore, excessive lipid oxidation can degrade the nutritional quality and reduce the shelf life of the product [46].

## 4. Conclusions

The physicochemical quality of rabbit burgers made from does of different ages remains unchanged during four months of frozen storage, and the chemical composition complies with current legislation. Based on the results obtained, it is recommended to use meat from rabbits of different ages for the preparation of burgers, as a way to optimize the use of breeding hens after the end of their production cycle, in addition to providing a greater economic return to producers by selling a product with added value to the consumer market.

## Figures and Tables

**Table 1:** Formulation of rabbit hamburger (%).

Hamburger (%)	
Rabbit meat	81.55
Chicken skin	10.00
Soy protein	3.55
Water	2.00
Salt	1.50
Antioxidant	1.00
Garlic paste	0.30
Ground black pepper	0.10
Total	100.00

**Table 2:** Mean (± SEM) values of luminosity (L*), redness intensity (a*), and yellowness intensity (b*) of burgers made with meat from Botucatu does of different ages (3, 12, and 24 months) stored at −18 °C for up to 120 days.

Variable	Animal Age (A)			Storage Time (T)			*p*-Value		
	3 Months	12 Months	24 Months	Beginning	60 Days	120 Days	*p* (A)	*p* (T)	*p* (A × T)
L*	71.7 ± 0.2 ^A^	70.9 ± 0.2 ^AB^	70.8 ± 0.2 ^B^	71.6 ± 0.2	71.0 ± 0.2	70.8 ± 0.2	0.021	0.053	0.801
a*	5.5 ± 0.1 ^B^	5.8 ± 0.1 ^A^	5.8 ± 0.1 ^A^	5.8 ± 0.1 ^A^	5.5 ± 0.1 ^B^	5.7 ± 0.1 ^AB^	0.007	0.049	0.379
b*	10.8 ± 0.3	10.8 ± 0.3	11.1 ± 0.3	10.0 ± 0.3 ^B^	11.7 ± 0.3 ^A^	11.0 ± 0.3 ^A^	0.691	<0.001	0.126

^A,B^ Means followed by different letters in the rows differ from each other by the Tukey test (*p* < 0.05). SEM: standard error of the mean; A: animal age; T: storage time; A × T: interaction of age and storage time of hamburgers.

**Table 3:** Means (± SEM) of cooking loss (CL), shrinkage percentage (SP), and weight loss during storage (SWL) of hamburgers made from meat of Botucatu does at different ages (3, 12, and 24 months), stored at −18 °C for up to 120 days.

Variable	Animal Age (A)			Storage Time (T)			*p*-Value		
	3 Months	12 Months	24 Months	Beginning	60 Days	120 Days	*p* (A)	*p* (T)	*p* (A × T)
**CL (%)**	43.70 ± 0.35 ^A^	42.02 ± 0.32 ^B^	42.05 ± 0.34 ^B^	43.10 ± 0.35	42.37 ± 0.34	42.35 ± 0.32	0.001	0.233	0.103
**SP (%)**	34.48 ± 0.87	34.78 ± 0.81	36.16 ± 0.81	36.10 ± 0.84	34.49 ± 0.81	34.83 ± 0.84	0.322	0.368	0.151
**SWL (%)**	10.42 ± 0.22 ^A^	9.34 ± 0.24 ^B^	10.40 ± 0.24 ^A^	-	10.27 ± 0.18	9.84 ± 0.18	0.001	0.114	0.103

^A,B^ Means followed by different letters in the rows differ from each other by the Tukey test (*p* < 0.05). SEM: standard error of the mean; A: animal age; T: storage time; A × T: interaction of age and storage time of hamburgers.

**Table 4:** Means (±SEM) of the interaction between age and storage time for pH and shear force of hamburgers made from meat of Botucatu does at different ages (3, 12, and 24 months) stored at −18 °C for up to 120 days.

Storage Time (T)	Animal Age (A)			*p*-Value	
	3 Months	12 Months	24 Months		
**pH**					
**Beginning**	4.68 ± 0.01 ^Bab^	4.66 ± 0.01 ^Bb^	4.69 ± 0.01 ^a^	*p* (A)	<0.001
**60 days**	4.73 ± 0.01 ^Aa^	4.70 ± 0.01 ^Ab^	4.70 ± 0.01 ^b^	*p* (T)	0.008
**120 days**	4.68 ± 0.01 ^B^	4.68 ± 0.01 ^B^	4.69 ± 0.01	*p* (A × T)	0.008
**Shear Force (N)**					
**Beginning**	8.11 ± 0.77	9.95 ± 0.74	9.71 ± 0.79 ^B^	*p* (A)	0.294
**60 days**	9.44 ± 0.79	8.53 ± 0.74	10.35 ± 0.82 ^AB^	*p* (T)	0.001
**120 days**	8.24 ± 0.79 ^b^	9.46 ± 0.76 ^b^	12.92 ± 0.82 ^Aa^	*p* (A × T)	0.044

^A,B, a,b^ Means followed by different capital letters in the columns and different lowercase letters in the rows differ significantly from each other according to the Tukey test (*p*< 0.05). SEM: standard error of the mean; A: animal age; T: storage time; A × T: Interaction of age and storage time of hamburgers.

**Table 5:** Means (±SEM) of the values for protein, mineral matter (MM), and lipids of hamburgers made from meat of Botucatu does at different ages (3, 12, and 24 months) stored at −18 °C for up to 120 days.

Variable	Animal Age (A)			Storage Time (T)			*p*-Value		
	3 Months	12 Months	24 Months	Beginning	60 Days	120 Days	*p* (A)	*p* (T)	*p* (A × T)
**Protein (%)**	21.72 ± 0.36 ^B^	24.04 ± 0.35 ^A^	22.92 ± 0.36 ^AB^	23.41 ± 0.36	22.84 ± 0.35	22.43 ± 0.36	<0.001	0.163	0.747
**MM (%)**	2.21 ± 0.03 ^A^	2.22 ± 0.03 ^AB^	2.14 ± 0.03 ^B^	2.18 ± 0.03	2.24 ± 0.03	2.21 ± 0.03	0.04	0.486	0.524
**Lipids (%)**	10.81 ± 0.29	10.78 ± 0.28	11.09 ± 0.28	9.98 ± 0.28 ^B^	11.73 ± 0.29 ^A^	10.98 ± 0.28 ^A^	0.691	<0.001	0.126

^A,B^ Means followed by different letters in the rows differ from each other by the Tukey test (*p*< 0.05). SEM: standard error of the mean; A: animal age; T: storage time; A × T: interaction of age and storage time of hamburgers.

**Table 6:** Means (±SEM) of the interaction values between age and storage time for moisture and lipid oxidation (TBARS) of hamburgers made from meat of Botucatu does at different ages (3, 12, and 24 months) stored at −18 °C for up to 120 days.

Storage Time (T)	Age Animal (A)			*p*-Value	
	3 Months	12 Months	24 Months		
**Moisture (%)**					
**Beginning**	64.52 ± 0.15 ^a^	62.97 ± 0.17 ^b^	63.36 ± 0.20 ^Bb^	*p* (A)	<0.001
**60 days**	64.41 ± 0.15 ^a^	63.29 ± 0.15 ^b^	64.24 ± 0.15 ^Aa^	*p* (T)	0.029
**120 days**	64.13 ± 0.15 ^a^	63.38 ± 0.15 ^b^	64.19 ± 0.15 ^Aa^	*p* (A × T)	0.010
**TBARS (mg MDA/kg)**					
**Beginning**	1.06 ± 0.05 ^Ba^	0.73 ± 0.05 ^Cb^	0.91 ± 0.05 ^Bab^	*p* (A)	<0.001
**60 days**	1.55 ± 0.05 ^Aa^	0.99 ± 0.05 ^Bb^	1.05 ± 0.05 ^ABb^	*p* (T)	<0.001
**120 days**	1.49 ± 0.05 ^Aa^	1.31 ± 0.05 ^Aab^	1.18 ± 0.05 ^Ab^	*p* (A × T)	<0.001

^A,B, a,b^ Means followed by different capital letters in the columns and different lowercase letters in the rows differ significantly from each other according to the Tukey test (*p* < 0.05). SEM: standard error of the mean; A: animal age; T: storage time; A × T: interaction of age and storage time of hamburgers.

## Data Availability

The data that support the findings will be available from Repositorio Institucional da UNESP at https://hdl.handle.net/11449/251659 (accessed on 1 May 2025) following an embargo from the date of publication to allow time for the article to be published first.
